# Obtaining Reliable RT-qPCR Results in Molecular Diagnostics—MIQE Goals and Pitfalls for Transcriptional Biomarker Discovery

**DOI:** 10.3390/life12030386

**Published:** 2022-03-07

**Authors:** Christian Grätz, Maria L. U. Bui, Granit Thaqi, Benedikt Kirchner, Robert P. Loewe, Michael W. Pfaffl

**Affiliations:** 1 Department of Animal Physiology and Immunology, School of Life Sciences, Technical University of Munich, Weihenstephaner Berg 3, 85354 Freising, Germany; chris.graetz@tum.de (C.G.); marialan.bui@tum.de (M.L.U.B.); granit.thaqi@mytum.de (G.T.); benedikt.kirchner@wzw.tum.de (B.K.); 2 GeneSurge GmbH, Ottostr. 3, 80333 München, Germany; robert.loewe@genesurge.com

**Keywords:** MIQE, transcriptional biomarkers, biomarker signature, RT-qPCR, molecular diagnostics

## Abstract

In this review, we discuss the development pipeline for transcriptional biomarkers in molecular diagnostics and stress the importance of a reliable gene transcript quantification strategy. Hence, a further focus is put on the MIQE guidelines and how to adapt them for biomarker discovery, from signature validation up to routine diagnostic applications. First, the advantages and pitfalls of the holistic RNA sequencing for biomarker development will be described to establish a candidate biomarker signature. Sequentially, the RT-qPCR confirmation process will be discussed to validate the discovered biomarker signature. Examples for the successful application of RT-qPCR as a fast and reproducible quantification method in routinemolecular diagnostics are provided. Based on the MIQE guidelines, the importance of “key steps” in RT-qPCR is accurately described, e.g., reverse transcription, proper reference gene selection and, finally, the application of automated RT-qPCR data analysis software. In conclusion, RT-qPCR proves to be a valuable tool in the establishment of a disease-specific transcriptional biomarker signature and will have a great future in molecular diagnostics or personalized medicine.

## 1. Introduction to Transcriptional Biomarkers in Molecular Diagnostics

Accurate and fast diagnosis is critical for a successful treatment of any disease. Traditionally, diagnosis and therefore treatment have been decided based on observation of specific patterns of symptoms in the patient. However, since many diseases are accompanied by similar and ergo unspecific implications, the consensus was reached that symptomatic diagnosis and treatment might not always be the best choice for a number of diseases. Thus, the focus has shifted toward the exploration of biological markers (biomarkers) in patient samples, which reveal distinct biological traits or changes in the organism and can therefore be connected to specific disorders [1]. Those biomarkers can mainly be found in the genome, transcriptome, proteome or metabolome and are defined as *“measurable indicator[s] of normal biological processes, pathogenic processes or pharmacological reaction to a therapeutic intervention”* [2]. The process of analyzing the presence or absence of biomarkers in clinical samples, aiming to identify a certain disease in the patient, is called molecular diagnostics. Along with new detection and quantification technologies, this field has been subject to rapid growth and sophistication in recent years. Nowadays, biomarker families can be detected and quantitatively measured in all kind of solid or liquid biological samples, even in very low concentrations [3,4]. Highly specific and sensitive molecular biology techniques provide significant assistance in molecular medicine, disease diagnosis and prognosis, as well as in agriculture and food safety.

Liquid biopsies have recently become more and more popular in molecular diagnostic research, with some assays having already been approved by health authorities [5]. Being minimally invasive, they promise accurate diagnosis with a sampling procedure that is easy to handle and reduces the risk of severe complications compared to conventional biopsies [6]. Furthermore, liquid biopsies can provide decision makers with a holistic overview of the disease status, including, e.g., metastases from tumor patients, and, consequently, systemic information that might be missing in tissue biopsies [5,7]. Blood plasma, as a prominently used sample, is advantageous in cases where, e.g., the patient cannot provide a tissue sample [8] or in order to detect early stages of disease not manifested yet in tissue [9]. Another well-used source for liquid biopsy is urine, e.g., for detection of endometrial cancer or bladder cancer [10,11], and saliva can be of diagnostic value for indication of pain, periodontitis and Alzheimer’s disease [12,13,14].

So far, proteins have been used predominantly as biomarker molecules, since they are present in relatively high concentrations in blood and allow for relatively cheap and fast detection in liquid biopsies by reliable and widely available antibody-based methods, such as enzyme-linked immunosorbent assay (ELISA) or immunohistochemistry (IHC) [15]. Furthermore, proteins are the biomolecules that facilitate most cellular processes or reactions and therefore provide direct information about a cell or tissue [16]. However, body fluids used in liquid biopsies, such as blood or urine, usually contain several other components that may also be utilized as disease biomarkers, e.g., cellular nucleic acids, circulating tumor cells (CTCs), extracellular vesicles (EVs) or circulating nucleic acids, such as circulating cell-free DNA (ccfDNA), circulating tumor DNA (ctDNA) and cell-free RNA (cfRNA) [6,17,18]. In fact, nucleic acids offer multiple advantages as biomarkers compared to proteins. For instance, the detection limits of DNA and RNA are significantly lower than those of proteins, since they can easily be amplified by polymerase chain reaction (PCR) or reverse transcriptase PCR (RT-PCR), respectively [15,19,20]. Additionally, nucleic acid detection is even more cost efficient than protein detection, without the need for highly affine and expensive antibodies. Furthermore, many diseases are directly caused by DNA changes, e.g., point mutations or DNA rearrangements, which can easily be detected by PCR-based methods and next-generation sequencing (NGS) but are significantly harder to measure via antibody-based methods.

In this review, we focus on molecular biomarkers on the transcriptional level. The transcriptome can be divided into RNA subtypes with distinct functions.

Messenger RNA (mRNA) carries the genetic sequence in a form that can be recognized to synthesize proteins. Since its expression frequently correlates with the type and amount of proteins produced, it is often directly linked to pathological processes and, therefore, a popular target for transcriptional biomarker studies. Furthermore, it is one of the most easily accessible biomarkers in liquid biopsy, as its expression in blood is 80% identical to that of major tissues [21,22]. However, only 1–2% of the human transcriptome encodes for proteins and is therefore transcribed to mRNAs [23]. The remaining 98% of the transcriptome consists of a multitude of non-coding RNA families, which can be divided into two groups based on their length: small and long non-coding RNAs.

Long non-coding RNAs (lncRNAs) include RNA transcripts longer than 200 nucleotides that do not encode for proteins. In the study performed by the consortium GENCODE, the human genome annotation consists of 15,512 lncRNA transcripts grouped in 9640 loci [24]. According to lncRNA location with respect to protein-coding genes, these lncRNA can be classified into intergenic lncRNAs, antisense lncRNAs and intronic lncRNAs [25]. Biological functions of lncRNAs are carried out through interaction with proteins, RNA or DNA and include transcriptional regulation, competing endogenous RNA, splicing regulation, translational control, protein localization and RNA interference [26,27]. The ability of lncRNAs to interact with different molecules in a wide range of biological processes underlines the necessity for a better understanding of their function and hence their application as biomarkers. Moreover, lncRNAs show more tissue-specific expression than protein-coding genes [24], and their functions are closely associated with a number of diseases [26,28]. Recently, there has been increasing evidence of lncRNAs as potential prognostic and diagnostic biomarkers, especially in cancer research [29]. For instance, H19—one of the first identified lncRNAs—is a biomarker for tumors of the esophagus, liver, bladder, colon and metastases in the liver [30,31,32,33]. Additionally, lncRNAs from blood biopsies could be established as biomarkers for the diagnosis of lung cancer not only in comparison to healthy controls but also in contrast to pneumonia [34].

Short non-coding RNAs (sncRNAs) include RNA transcripts of fewer than 200 nucleotides in length, such as ribosomal RNA (rRNA), transfer RNA (tRNA), small interfering RNA (siRNA), small nucleolar RNA (snoRNA) and, most importantly for biomarker research, micro-RNA (miRNA) [28]. miRNAs prevent their respective target mRNAs from translation into proteins or accelerate their degradation primarily by binding to their 3′ UTR or alternatively in the protein-coding region. They are abundant in many mammalian tissues, and only a small portion of them are housekeeping miRNAs [35]. Due to their short length of about 22 nucleotides, they are more resistant to RNase exposure and more stable than mRNA [36]. miRNAs can be found in any body fluids, such as urine, blood, sweat, saliva and milk [37,38,39], where they are protected from RNase degradation, e.g., by extracellular vesicles or RNA-binding proteins. Hence, some of those circulating miRNAs were shown to serve as optimal biomarkers for several types of cancer [40,41]. Fueled by the development of high-throughput sequencing, the identification of miRNA biomarkers is progressing steadily, and the newly achieved levels of sequence resolution enabled the discovery of miRNA isoforms, so-called isomiRs [42]. isomiRs differ from canonical miRNAs in length and minor sequence substitutions [43]. Depending on their alteration, they can target mRNAs in cooperation with their canonical miRNAs or gain a completely new target repertoire [44,45,46,47]. Moreover, isomiRs display even higher discriminatory power than canonical miRNAs for a large number of cancer types [48]. The exploration of miRNA biomarkers is still an emerging field, and more insights might be gained by studying miRNAs at the isomiR level [43]. Table 1 provides an overview over different RNA types and their potential use as biomarkers.

Transcriptional biomarkers, in contrast to DNA and proteins, allow for immediate detection of cellular changes [15]. The transcriptional profile of a cell responds to a signal within minutes, while the alterations are only visible after hours on protein level. In the DNA, on the other hand, these changes are harder to detect, as they are usually regulated via methylation or binding of transcription factors. Furthermore, the amount of RNA in a cell exceeds that of DNA by far. Consequently, the copy number of a specific nucleic acid biomarker sequence in blood and other body fluids is much higher and thus easier to detect on RNA than on DNA level [15]. This effect is even more prominent in diseases where certain genes are overexpressed, e.g., HER2-positive breast cancers [49]. However, proteins remain the most widely used type of biomarkers in diagnostics, and the use of transcriptional biomarkers in routine diagnostics is still very limited. Nevertheless, their vast potential has been recognized by researchers, and there is a growing number of publications developing new nucleic acid biomarker signatures for various diseases [49,50,51,52,53,54,55]. Table 2 shows a comparison of different types of biomarkers and compares the information they provide along with their advantages and disadvantages.

**Table 1:** Overview of reported biomarkers from different RNA types and their potential use. Abbreviations: isomiR (miRNA isoforms); lncRNA (long non-coding RNA); NGS (next-generation sequencing); mRNA (messenger RNA); miRNA (micro-RNA); piRNA (piwi-interacting RNA); RNA-Seq (RNA sequencing); RT-qPCR (reverse transcription quantitative real-time polymerase chain reaction); siRNA (small interfering RNA); snoRNA (small nucleolar RNA).

RNA	Sample Type	Disease	Expression Level	Potential Use
**mRNA**				
PON2 (Paraoxonase-2) [56]	Tissue, urine, cell lines	Bladder cancer	Upregulated	Diagnosis
PAM50 [15]	Tissue	Breast cancer	Upregulated	Diagnosis/ Prognosis
mRNA tHERT [57]	-	Acute myeloid leukemia	-	Therapeutics
**lncRNA** **(>200 nt)**				
XLOC_009167 [34]	Whole blood	Lung cancer	Upregulated	Diagnosis
HOTAIR [58]	Breast epithelial cells	Breast cancer	Upregulated	Prognosis
**miRNA (~22 nt)**				
miR-421 [59]	Carcinoma tissue	Gastric carcinoma	Upregulated	Diagnosis
miR-141 [60,61]	Plasma	Prostate cancer	Upregulated	Diagnosis
**isomiR (~22 nt)**				
5’ísomiR-140-3p [44]	Cancer tissue, cell lines	Breast cancer	Upregulated	Prognosis
miR-574-3p (3´ deletion A) [62]	Serum	Esophageal squamous cell carcinoma	Upregulated	Diagnosis
**piRNA (26–31 nt)**				
piR-1245 [63]	Tissue	Colorectal cancer	Upregulated	Prognosis
**snoRNA (60–300 nt)**				
SNORA17A [64]	Tissue	Hepatocellular carcinoma	Downregulated	Prognosis
**siRNA (19–23 nt)**				
ALN-TTR02 [65]	-	Familial amyloidotic polyneuropathy	-	Therapeutics

As will be elaborated in the following chapters, the establishment of RNA-based biomarkers starts with RNA-Seq, which gives a broad and holistic overview over potential candidate genes. These will be subsequently validated with RT-qPCR, which is ideally subjected to the MIQE standards. RT-qPCR is the method of choice for evaluation of candidate RNA biomarkers, due to its sensitivity in detection of less frequent biomarkers, as well as its specificity.

## 2. From Biomarker Development to Routine Diagnostics

### 2.1. RNA-Seq for Holistic Screening and Development of the Biomarker Signature

The first step toward developing a transcriptional biomarker signature for a specific disease is holistic RNA screening of a training cohort. RNA-Seq is the method of choice for this purpose, since it allows the measurement of the RNA expression of all genes present in the sample. In many cases, there is not enough prior knowledge of potential RNA sequences linked to the disease of interest or whether they can be found in the chosen sample type to limit the expression analysis to only one RNA subset in the sample. Even if possible biomarker RNAs are already known, it is recommended to perform a holistic screening instead of a targeted expression analysis, since those candidate RNAs might not be the best suited biomarkers in the sample type of choice. Most diseases affect multiple cellular pathways and thereby the expression of a vast number of different RNAs. Some of them might be selectively enriched or depleted in the analyzed samples, either biologically or technically, e.g., by potentially selective sorting into EVs or by the choice of isolation method [71,72,73].

The training cohort contains isolated RNA from patients diagnosed with the disease of interest, along with RNA from a control group, i.e., healthy volunteers, and is used to generate a cDNA library for sequencing. The sample type from which the RNA is isolated always depends on the RNA type of interest and the samples typically available for the specific disease—it should be the same as the one that will be used later in routine diagnostics. In liquid biopsy research, serum might be used to develop an EV-associated miRNA signature for community-acquired pneumonia (CAP) and pneumonia-related sepsis [50]. In cancer biomarker research, for instance, either tissue or blood samples might be used. Tissue samples are often routinely taken for clinical diagnosis or during invasive surgery, e.g., punch biopsies for breast cancer subtyping. Blood samples, on the other hand, combine information and provide an averaged view of the whole body and from all organs, including pathological metastases in cancer patients. Biomarkers for some organ-specific diseases can also be found in more easily accessible samples than tissue biopsies and still be specific to a small subset of organs only. For example, several potential biomarkers for bladder cancer have been found in urine [74]. Thus, the choice of sample type and matrix used for transcriptional biomarker development has an enormous impact on the significance and informative value of the possible results and should be well thought out prior to the study.

Another important step to consider is the method used to isolate RNA from the sample. After all, RNA isolation should be comparatively quick, easy and cost efficient for transcriptomic biomarkers to compete with already-established methods in routine diagnostics. On the other hand, a high RNA yield is crucial; otherwise, important transcripts with essential information might get lost. The studied disease, as well as the sample type chosen, directly influences which type of RNA is most promising as a biomarker. For instance, mRNAs overexpressed in certain cancers are a good target for biomarker studies in tissue samples. Since the RNA found in blood—especially cfRNA—might be partly degraded, small RNAs, such as miRNA, are usually investigated when working with blood samples or cfRNA. Consequently, the RNA type chosen as a possible biomarker source also affects the choice of isolation method, since different isolation strategies might enrich different RNAs [15,71,75,76,77]. For EV-associated RNA and cfRNA, the method used to isolate EVs from the sample has also been shown to impact RNA-Seq results [72], highlighting the importance of this aspect regarding biomarker development. In general, an RNA quality evaluation control step should be introduced after RNA isolation, assessing the degradation status (e.g., by RIN) and yield of isolated RNA from each sample. Afterward, a standardized amount of RNA—and only from samples showing comparable RIN numbers and RNA quality—should be used for sequencing.

To reduce possible batch effects, all samples from the training cohort should be handled simultaneously during library preparation and sequenced in the same run. However, library preparation is a crucial step that is prone to introduce technical bias in RNA-Seq experiments [78,79,80,81], even when all samples are handled the same way. Possible pitfalls are rRNA depletion, fragmentation, reverse transcription (RT), PCR amplification and size selection, but the greatest bias in library preparation is possibly introduced by the ligation of adapters and barcodes to the RNA fragments [78,80]. To avoid the ligation bias, at least for the barcode, it is strongly recommended to introduce it only in a later step, e.g., RT or PCR [78]. Compared to the library preparation protocol, the choice of sequencing platform does not seem to impact RNA-Seq findings as much [81,82].

After next-generation sequencing, the results are processed in silico. The steps vary with the length and type of the analyzed RNAs and include filtering, trimming and mapping against a suitable database for the analyzed type of RNA. Obviously, the parameters used for filtering depend on type and size of the analyzed RNAs and have a big impact on the results. Using filtering parameters that are too broad, sequencing artifacts and low-quality sequences will remain in the data set, while overly strict parameters might eradicate some potential biomarkers. Applying multivariate statistics, e.g., hierarchical clustering (HCA), principal component analysis (PCA) or partial least-squares discriminant analysis (PLS-DA) to the processed data, a subset of RNA genes is identified, which are up- or down-regulated in the treatment compared to the control group samples [3,83,84]. PCA is a powerful and popular statistical method often used to establish potential biomarkers from processed RNA-Seq, which reduces the multidimensional data set down to two or three parameters. These remaining projected dimensions are called principal components. They help to visualize clusters in the data for human eyes in a simple two- or three-dimensional plot. Analyzed samples are ranked in the result plot according to the amount of variance they explain [83,85]. Therefore, RNA genes contributing to the separation of samples in PCA are promising biomarker candidates [78]. However, PCA as an unsupervised clustering method does not incorporate any technical information about the experiment. This means that the highest ranked principal components may correspond to experimental setup, such as different sampling or batch effects, consequently masking information about potential biomarker genes. To avoid this limitation of PCA, a supervised classification method, for example (sparse) PLS-DA, may be used instead [84].

The obtained preliminary signature of transcriptional biomarkers, derived from the first learning cohort of patients, is then validated with more samples in the second validation cohort using an orthologous method. Since the number of RNAs that are analyzed in the validation cohort has been reduced to a smaller subset of biomarker candidates, a more time- and cost-efficient method can be used. RT-qPCR is a well-established and—even more relevant—standardized method to confirm and validate the candidate biomarker signatures when in compliance with the MIQE guidelines, as was shown by several recently published studies [20,86,87,88].

### 2.2. RT-qPCR for Confirmation and Validation of the Candidate Biomarker Signature

To validate the biomarkers found by RNA-Seq in the learning cohort, RT-PCR primers have to be designed first. Primers provide the binding site for the polymerase and act as a starting point for amplification of the RNA of interest via a hydroxyl group at their 3′ end. This primer design step is the basis for the following biomarker validation and should be given appropriate thought, since the specificity and efficiency of any PCR assay are critically dependent on the primers. Suitable primers are usually found with publicly available software, e.g., Primer3 [89]. Important parameters to consider are primer length, melting temperature (T_m_) and amplicon size, among others. Repeats and runs of bases in the primer sequence, unspecific primers, as well as the formation of secondary structures, should be avoided [90,91]. Since biomarker candidate sequences must be normalized against reference genes in order to make them comparable between different samples, it is especially important that all RT-PCR assays possess a comparable amplicon size [91]. Otherwise, effects such as (partially) degraded RNA will distort the results. When validating mRNA biomarker candidates, the amplicon should contain an exon–exon junction to ensure primers are RNA specific and avoid genomic DNA (gDNA) amplification. All primer sequences and their exact binding sites should be reported to comply with the MIQE guidelines, even when using commercial primers [90].

In order to amplify transcriptional biomarkers, the RNA of interest needs to be reverse transcribed from RNA to cDNA. Since reverse transcription is a major source of variability between RT-qPCR experiments, it should ideally be performed in two or three technical replicates, preferably at the RT level [90]. To avoid the resulting additional workload, spike-in controls might be used instead to assess RT efficiency [92].

For every assay, all samples should be measured in the same PCR run to ensure inter-sample comparability. Additionally, it is recommended to include an inter-run calibrator (IRC) on each plate, so the different assays can be quantified relative to each other [92].

Biomarker candidates are first validated in the same training cohort that was used in RNA-Seq. To ensure only specific PCR products are measured, a melting curve analysis must be performed when using intercalating dye-based qPCR. While a single peak in the melting curve indicates the presence of a single, specific PCR product, multiple peaks can be indicative either of a multi-stage melting transition (caused by, e.g., secondary structures or GC-rich regions) or of unspecific PCR products. In case of a preponderance of unspecific PCR products, primer design must be resumed and optimized. Additionally, potential outliers may be removed before further analysis, such as single wells containing products with incorrect melting temperatures [92]. The procedure for data exclusion must be well documented, justified and reported to avoid misinterpretation of data. Subsequently, the authenticated C_q_ values for each biomarker assay are normalized against reference genes, resulting in a ΔC_q_ value [93]. These reference genes must be chosen carefully depending on the RNA and sample type, as further explained below. Then, the ΔC_q_ values of patient samples are subtracted from healthy volunteer ΔC_q_, yielding the ΔΔC_q_ value. Relative fold gene expression is then calculated using the formula: 2^−ΔΔCq^ [93]. A fold change in gene expression above a certain value—e.g., 2–5, depending on the amount of target being quantified—is considered to be significant for *p* values < 0.05 in a two-tailed Student’s *t*-test [92]. Finally, the biomarkers are validated a second time in an independent validation cohort in the same manner.

Although this approach might come along with additional laboratory and bioinformatical workload, as well as added costs regarding consumables and time [92], it is meaningful to apply RT-qPCR as a validation step for candidate biomarkers preselected via RNA-Seq. NGS is technically more demanding in direct comparison to RT-qPCR [94], and, therefore, the potential for errors is much higher. The increased complexity of NGS can also hamper the chances of reproducibility [95]. On the other hand, RT-qPCR can check for the selected candidate RNA biomarkers in biological replicates and can thus validate the RNA-Seq-data on a bigger sample size in a second additional independent patient cohort. Due to the preselected, smaller set of genes of interest, RT-qPCR is a more cost-effective and time-efficient method in contrast to the initial holistic RNA-Seq [96].

## 3. Why Is MIQE and Standardization in RT-qPCR So Important?

Since its introduction to the scientific world by Kary Mullis in 1985 [97], the polymerase chain reaction (PCR) made itself indispensable in the scientists’ toolbox through its sensitivity and specificity in detection of nucleic acids. Further developments of the technique resulted in second- and third-generation PCR, namely RT-qPCR and digital PCR (dPCR), adding simplicity and enhanced precision to the desirable features of the molecular method.

RT-qPCR is unparalleled as the “gold standard” of gene expression profiling [98] and detects both the presence and quantity of specific single-stranded nucleic acids (cDNA or RNA) in real time. However, a closer meta-analysis shows that many of the data generated through RT-qPCR are contradictory and of poor quality, and thus some conclusions might not be valid [99]. The lack of details on the experimental conditions further complicates the assessment of the validity of the experiments and their outcomes. This poses a problem and fuels the “reproducibility crisis” in science [100], since non-reproducible results bear the risk of distorting a topic and introducing a bias into the research direction, as published papers serve as the basis for further research and projects. The report of Ramsden et al. [101] shows that years after RT-qPCR entered the laboratories as an everyday method, there was still no general consensus on what a well-executed RT-qPCR should look like. This highlights the need for certain standards regarding planning, performing and analyzing RT-qPCR-based experiments in order to generate reliable results.

The international standardization rules for RT-qPCR were first laid down in 2009 in form of the Minimum Information for Publication of Quantitative Real-Time PCR Experiments (MIQE) guidelines, which were established by active researchers under the auspices of Minimum Information for Biological and Biomedical Investigations (MIBBI) [90]. Not only is molecular biology work in academia guided by the MIQE guidelines, but also major biotech companies in the field of gene quantification and clinical diagnostics abide by the guidelines.

According to the MIQE guidelines, the factors that will have an impact on the performance of RT-qPCR fall into three broad categories: (1) pre-PCR sample collection and handling, (2) PCR assay design and (3) post–PCR data analysis. Errors regarding the sample include inadequate sampling procedure, suboptimal buffer and storage conditions, inadequate sample preparation and, therefore, reduction in nucleic acid quality, purity and quantity. Presence of RT or PCR inhibitors in low-quality samples may lead to false negative results. Inhibitors can be detected using an internal amplification control or by diluting the sample and calculating the amplification efficiency, for example [102]. Alternatively, an assay detecting a spiked-in positive control, e.g., the SPUD assay, might be used [103]. Further errors in terms of assay design might be the selection of less specific primers, causing the assay to underperform. Finally, inadequate data analysis followed by misinterpretation can intentionally or unintentionally bias the results. Data analysis also involves wrong or missing controls, unreliable reference gene selection, exclusion of outliers, etc. Further potential sources of outcome variability are different data analysis methods of qPCR experiments [104], lack of maintenance of technical hardware, equipment being too outdated, no suitable plastic consumables [105], different consumables (e.g., buffer composition) or different methods to determine the quantity and quality of nucleic acids [106]. Pre-analytical and analytical parameters can impact the overall results for clinical biomarkers, such as ERBB2 (Her2), in multiple techniques. This was seen in detail by the impact of RNA integrity on subsequent analysis via RNA-Seq in breast cancer patients [107], as well as for additional pre-analytic parameters [108]. RNA isolation protocols and RNA integrity can also alter ERBB2 results in RT-qPCR experiments for such patient cohorts [109].

The need for a standardized study design using RT-qPCR as method is reflected in the number of scientific citations in peer-reviewed journals. As of today (December 2021), the first MIQE guidelines by Bustin et al. [90] have been cited over 8800 times, according to Web of Science. This version of standardization rules for RT-qPCR-based experiments has since been further developed; in 2013 a “good practice guide” for the application of dPCR was published and renewed in 2020 [110,111].

The authors of the MIQE guidelines aim to accomplish three things: (1) to provide a guide to help scientists design and evaluate meaningful qPCR experiments; (2) to provide editors and reviewers with evaluation criteria to better assess the quality of submitted studies/papers; and (3) to facilitate the reproduction of papers that have followed the MIQE guidelines. Compliance with MIQE guidelines generated only slightly higher costs, as described by Dooms et al. [112]. Standardization is essential, not only in research, but also in diagnostics, as will be elaborated below. However, eight years after the introduction of the first MIQE guidelines, most RT-qPCR-based studies still lack the most basic information on their study design [100]. Enforcing standards in RT-qPCR remains as urgent as ever for the sake of reproducibility in science and, furthermore, for translation in clinical settings, such as the development of biomarker signatures.

## 4. Examples for Application of RT-qPCR as a Fast and Reproducible Method in Routine Diagnostics

Not only research, but also diagnostics recognized the accuracy and reliability of RT-qPCR. Due to the method’s relative simplicity [113], laboratory personnel does not need extensive training to run this application, and in consequence, RT-qPCR is a method which can be—and already is—broadly implemented in routine diagnostic laboratories.

Liquid biopsy, as stated above, utilizes biofluids obtained with non- or minimal invasive techniques for diagnosis [114]. The relatively minor disturbance of the patient’s physical health allows for repeated sample collection to monitor the treatment over an extended period of time, as patient compliance is very high [115]. This is especially advantageous in oncology, either by analyzing the presence or absence of genes of interest, as well as their relative gene expression, or by screening for certain point mutations [116]. However, most diseases are rather determined through combined mutations in several genes. To give an example—in breast cancer, the expression patterns of *ERBB2*, *ESR1*, *PGR* and *MKI67* are used for subtyping the disease and to determine the treatment according to the St. Gallen guidelines (2017) [117]. Relative gene expression levels of these biomarkers can be assessed in RT-qPCR-based tests, such as OncotypeDX, EndoPredict, MammaPrint and Prosigna, to detect breast cancer in an early stage. In follow-up care after cancer treatment, patients can be monitored using RT-qPCR. With regard to minimal residual disease measurement, it is possible to screen the patient for traces of leukemic cells [118,119] and to determine the success of the treatment or to detect relapses or recurrences, therefore enhancing the survival chances of the patient. Under strict conditions, the aforementioned mamma carcinoma gene expression tests are already used post-treatment for prognosis of the risk of relapse in Germany [120].

Another field where RT-qPCR proves to be a valuable tool is infectious disease diagnostics. In this case, gene expression levels of pathogens are determined. For instance, monitoring of the viral load is imperative in HIV patients. One commercially available kit for this purpose is the AltoStar HIV RT-PCR Kit 1.5. Without doubt, the most prominent example for the diagnostic use of RT-qPCR is the currently ongoing SARS-CoV-2 pandemic. Samples processed with RT-qPCR yield results within a few hours to determine whether the patient is infected. This allows for (1) suitable treatment, when other illnesses, such as cold or flu, are excluded and (2) quick reactions to quarantine the patient and thus minimize the potential of spreading the disease. Interestingly, the pandemic fuels further developments to reduce the cost of RT-qPCR tests for the benefit of the broader public [121,122,123,124].

When using RT-qPCR for molecular diagnostics, one should pay careful attention to experimental design, sample and assay quality, especially with focus on the efficiency of the reverse transcription reaction, the selection of proper reference genes and how the normalization will be calculated in the applied data software tool. All those factors significantly impact the chance of successful and reliable relative gene expression results [125]. In the next paragraphs, we focus on three aspects having a major effect on the results of transcriptional biomarker studies: (1) the reverse transcription reaction, (2) the selection of the right reference genes in relative quantification and (3) that the selected software is calculating the correct relative quantities of the target genes.

## 5. Importance of Reverse Transcription in RT-qPCR

Reverse transcription (RT) is presumably the most crucial and variable step in the entire RT-qPCR workflow. For the quantitative measurement of any RNA family, the RNA is first reverse transcribed into complementary DNA (cDNA). Afterward, the cDNA is profoundly amplified by a DNA-dependent DNA polymerase during the PCR cycling process. However, even today, 50 years after the discovery of the reverse transcriptase enzyme [126], the reaction is not yet fully understood and remains one of the most uncertain steps in gene expression analysis. The RT can introduce errors, based on complex secondary and tertiary structures of long RNA fragments, variation in priming efficiency and enzymatic properties of the different reverse transcriptase enzymes themselves [127,128]. RT yield, reproducibility, sensitivity, accuracy and precision of the reverse transcription reaction of commercially available enzymes on various target genes have been tested in multiple studies, and the results were highly variable between enzyme type and reaction condition. The efficiency of the RT reaction varies up to 100-fold with the choice of reverse transcriptase enzyme. That variation is dependent on the gene, primers and the applied priming strategy. In conclusion, it is recommended to use the same priming strategy and reaction conditions in all experiments and the same total amount of RNA in all samples for comparable RT-qPCR gene expression measurements between studies or laboratories. Experimental accuracy is improved by running samples in a minimum of two technical replicates, starting with the reverse transcription reaction [127,128].

## 6. Proper Reference Gene Selection

Choosing the appropriate quantification strategy is a very important step of gene quantification and therefore required by the MIQE guidelines. Normally, two quantification strategies can be applied in the RT-qPCR: absolute and relative quantification. Absolute quantification creates a correlation between the quantification cycle (C_q_) and input copy number using a calibration (or standard) curve [129]. This calibration curve is then used to determine the concentration of the unknown nucleic acid analyte [129]. In contrast, the relative quantification strategy analyzes changes in gene expression levels in each sample relative to another reference gene. It is based on the relative expression of a target gene transcript to one or more endogenously expressed reference gene transcripts [130]. A reference gene can be described as a gene, which is constantly expressed under different biological conditions. Hence, in the early days of RT-qPCR these genes were named housekeeping genes. Genes encoding for GAPDH, albumin, actins, tubulins, cyclophylin, microglobulin, 18S RNA or 28S RNA are widely used in several studies as reference genes [131]. One well-established reference gene used in liquid biopsy from blood is *PGK1* [132]. However, it has been demonstrated that no reference gene is universally stable over all biological conditions and that a different set of genes with the least variance exists for every biological context [72,78]. As discussed by Vandesompele et al. (2019), the commonly used reference genes *GAPDH* and *ACTB* turn out to be less reliable than assumed in cancer-related research. Underlying mechanisms explaining this observation might be the heterogeneity and genetic instability of cancerous tissue [133]. Therefore, in some cases, the classical reference genes are not stably expressed throughout all analyzed samples. On the contrary, reference genes may even be regulated in some diseases, and thus universally stable reference genes do not exist [130]. Caution must be used when selecting reference genes, and it must be assured that they are stably expressed in the sample type of interest. Unfortunately, reference genes are often selected without prior verification and independent consideration of the biological context [130,134,135,136], although different molecular diagnostic technology providers offer various panels with numerous reference genes to check for the most stably expressed reference genes.

For research purposes, software tools have been developed to identify stably expressed genes in a cohort. Convenient algorithms and software tools that analyze expression data obtained through any quantitative method to select valid reference genes include geNorm [130], BestKeeper [137], NormFinder [138], RefGenes [139] or miREV [140]. geNorm, so far the most popular algorithm for this purpose, ranks the reference genes based on the relative variation of pairwise expression values of the given samples. This variability is a so-called M-value; the higher the M-value, the more stably expressed the gene is [130]. BestKeeper can compare expression levels of up to ten reference genes together with ten target genes to determine the most appropriate standard genes among them, and it combines them into one index. This index can then be compared to other target genes to determine their expression levels in the given treatment [137]. NormFinder ranks the group of candidate normalization genes based on the global average expression of all genes in all samples, which are compared to individual genes. From this comparison, a standard deviation (SD) for each sample is generated. The genes with the lowest SD are the most reliable reference genes. Moreover, if different treatments are used, NormFinder can separate the variations into an intragroup and an intergroup contribution [138]. RefGenes is an online application that allows users to search for genes with stable expression in a selected sample set based on microarray data. This sample set can be selected according to experimental conditions or tissue types [139]. Recently, numerous studies have demonstrated the potential of RNA associated with extracellular vesicles (EVs), mainly miRNA, as biomarkers [48,50,71,72]. Online tools and databases such as miREV enable the user to find stably expressed miRNAs from EV studies regarding different experimental conditions. miREV includes data sets from publicly accessible sources, focused on blood-derived EVs and represents nine different pathologies and three different isolation methods from serum and plasma [51].

## 7. Automated RT-qPCR Data Analysis Software

Besides the classical RT-PCR parameters, e.g., primer design, RNA quality, RT and polymerase performances and the selection of reference genes—as discussed above—the fidelity of the quantification process is highly dependent on valid data analysis. The choice of analysis method is an important topic and must also be MIQE compliant [90]. Algorithms and software tools selected for this purpose should be robust, reproducible and reliable to generate valid results. As explained in detail above, transcriptional markers identified by RNA-Seq and multivariate biostatistical methods can be combined in a biomarker signature of relevant transcripts. The final goal for the RT-qPCR results, in order to validate this biomarker signature, is to reflect the findings of the RNA-Seq discovery study. Hence, a successful application of RT-qPCR for transcriptional biomarker development and post-PCR data processing depends on a clear understanding of the pitfalls in relative quantification methods.

Facilitating easy data management and providing tools for automated data analysis to obtain statistically proven results are the main goals in RT-qPCR data processing and application software development. All of the calculation and statistical software applications described were already summarized and discussed in other publications [141]. However, we should realize that post-qPCR data processing can influence or even change the final results of gene expression analysis. Events such as RT-qPCR data generation, acquisition, evaluation, calculation and statistical analysis, are essential to interpret the biological significance of an experiment. Depending on the type of data and the biological question, RT-qPCR data analysis might include curve fitting algorithms, data processing, selecting or discarding certain data subsets based on specific pre-set criteria, transformation of logarithmic C_q_ values to relative quantities, normalization, rescaling and a final statistical test of the derived qPCR results.

Accurate and straightforward mathematical and statistical analysis of post-PCR data and management of growing data sets have become the major hurdles for effective implementation into gene expression analyses. Nowadays, high-throughput 384-well applications generate huge amounts of RT-qPCR data, which in turn need to be grouped, standardized, normalized and documented by the software applications [142]. However, the cycler hardware, as well as the performance and chemistry, have developed much faster in the past than the post-PCR analysis software. Developing a “one-fits-all” software for simple and reliable analysis of the generated expression data would appear to be the optimal solution to obtain valid and comparable results from enormous amounts of data. However, specific demands and biological questions are too heterogeneous to implement this into a single software tool. The majority of RT-qPCR users analyze their relative expression data in the software tools provided with the cycling platform. Besides those, only a few independent and freely available software tools that still fulfill the stringent MIQE recommendations have survived in the scientific community. Out of these, the three most used tools for reliable, efficiency-corrected and reference-gene-normalized calculation of relative gene expression are GenEx [143], qBase+ [142] and REST [144].

GenEx [143] is a software tool developed by a Swedish group, which provides a multitude of functionalities for the RT-qPCR community in research and clinical molecular diagnostics. Various applications are included, e.g., data pre-processing and management or advanced cutting-edge multivariate analysis of big RT-qPCR data sets. It provides various methods for the selection and validation of reference genes, as described above. Besides qPCR data analysis, it can also cope with small data sets for RNA-Seq gene expression profiling. Furthermore, the software package contains a wide range of multivariate statistical and machine-learning algorithms, which makes it an optimal bioinformatical tool for gene expression data analysis and to find reliable transcriptional biomarker signatures.

The comprehensive software application qBase and the newer version qBase+ were developed as a generalized solution to accommodate virtually all relative quantification setups and for the management and automated analysis of real-time quantitative PCR data [142]. It employs a well-proven ΔC_q_ quantification model with efficiency correction, multiple reference gene normalization and accurate error propagation along all calculations. The qBase+ browser allows data storage and annotation while keeping track of all real-time PCR runs by hierarchically organizing data into projects, experiments and runs. As a big advantage, there is no limit on the number of samples, genes or replicates, and data from multiple runs can be combined and processed together. Furthermore, the ability to use up to five reference genes allows for reliable and robust normalization of gene expression levels on the basis of the geNorm procedure [130].

The relative expression software tool (REST), for comparison of several expressed genes, is based on Microsoft Excel and programed in the Visual Basic Application (VBA). The underlying mathematical model is based on the mean C_q_ deviation of target genes between the treatment and control group, normalized by multiple reference genes and PCR efficiency-corrected [144]. Subsequently, all relative expression data are statistically tested by a Pair-Wise Fixed Reallocation Randomization Test [144].

## 8. Summary and Conclusions

The potential pitfalls mentioned above highlight how important it is to carefully consider every step in a transcriptional biomarker discovery and development study—from the initial RNA-Seq screening to data analysis in clinical practice. Through suboptimal sample choice or mistakes during the RNA-Seq workflow, one might overlook promising biomarkers. Careless primer design and neglect of the MIQE guidelines in the RT-qPCR experiment may lead to the wrongful validation of transcripts, insufficient as a measure for the disease of interest. Finally, faulty data evaluation might result in further misinterpretation of the obtained information and—in the worst case—publication of an incorrect biomarker signature. Therefore, having an overview of the advantages and pitfalls of each method used is critical to avoid drawing false conclusions from the acquired data. In the context of liquid biopsy, RNA is a suitable analyte to discover and monitor the course of disease, as well as the progress of the patient’s treatment. RNA is excessively more abundant than DNA; its detection level is significantly lower than that of proteins, and the potential of different RNA classes in research and diagnostics as biomarkers is constantly expanding. Discovering a set of disease-specific candidate genes is performed via hypothesis-free RNA-Seq, using a training cohort consisting of patient samples. The chosen RNA type, as well as extraction method, must be given appropriate thought to enrich high-quality nucleic acids for sequencing. After using suitable parameter filters, the resulting set of candidate genes should be validated using an orthologous method, such as RT-qPCR, both in the initial training cohort and an additional independent cohort. Abiding by international standardization rules ensures the accuracy and validity of RT-qPCR-based results. Subsequently, in a translational approach, the validated genes of interest can be incorporated into diagnostics. Again, RT-qPCR, as the method of choice for gene expression analysis, is guided by the MIQE guidelines to reinforce transparency of the experimental setup for the sake of reproducibility. Possible pitfalls are present in sample handling, primer design and post-PCR data analysis. One equally important factor to consider is the appropriate choice of reference genes, for which publicly available software tools provide extensive support. Post-PCR data processing and statistical analysis have a major impact on the expression profiling results of transcriptional biomarkers, and the development of different automated RT-qPCR data analysis software is aimed to answer distinct biological questions.

In summary, RT-qPCR—when used in compliance with the MIQE guidelines—proves to be a valuable tool in the establishment of disease-specific biomarkers and in clinical setting, since it is an accurate and fast method with relatively simple handling. RT-qPCR for molecular diagnostics, particularly with regard to personalized medicine, is well positioned to pave its way into daily clinical routine in the near future. It is therefore all the more important to have and abide by standardized procedures for every step involved.

## Figures and Tables

**Table 2:** Comparison of different biomarker types, along with the information they contain, the most widely used detection method and their advantages and disadvantages. Abbreviations: ccfDNA (circulating cell-free DNA); CTC (circulating tumor cells); ELISA (enzyme-linked immunosorbent assay); EV (extracellular vesicle); FISH (fluorescence in-situ hybridization); IHC (immunohistochemistry); NGS (next-generation sequencing); qPCR (quantitative polymerase chain reaction); RNA-Seq (RNA sequencing); RT-qPCR (reverse transcription quantitative real-time polymerase chain reaction).

Type of Biomarker	Sample Type	Information	Detection Methods (i.a.)	Advantages	Disadvantages
Proteins [16]	Tissue biopsies	Expression pattern in tissue of interest	ELISA IHC Immunoblotting Flow Cytometry	Direct mediators of cellular changes Well-established biomarkers and assays available	Expensive antibody-based detection In most cases invasive sampling necessary
	Liquid biopsies (body fluids): blood, urine, saliva, i.a.	Systemic expression information	ELISA IHC Immunoblotting Flow Cytometry	Direct mediators of cellular changes High-throughput methods available Minimal invasive sampling	Expensive antibody-based detection Tissue of origin not determined
Intracellular RNA [15,66]	Tissue biopsies	Expression pattern in tissue of interest	RT-qPCR RNA-Seq Microarrays FISH	Expression-level analysis Detection of ncRNA	In most cases invasive sampling necessary
	Liquid biopsies: blood	Systemic expression information	RT-qPCR RNA-Seq Microarrays	Expression-level analysis Immunophenotyping Analysis of ncRNA Minimal invasive sampling	Tissue of origin not determined Almost exclusively information about immune response
Extracellular RNA, e.g., EV-associated [15,67,68]	Liquid biopsies (body fluids): blood, urine, saliva, i.a.	Systemic expression information	RT-qPCR RNA-Seq Microarrays	Semi-direct information Analysis of ncRNA Minimal invasive sampling	Fragmented Relatively new (few biomarkers and assays established) Only partial transcriptome Tissue of origin not determined
Genomic DNA [8,69]	Tissue biopsies	Mutations in the tissue of interest Epigenetic status in tissue of interest	qPCR NGS	Complete genome of the tissue of interest	Only indirect expression information available In most cases invasive sampling necessary
	Liquid biopsies (body fluids): blood, urine, saliva, i.a.	Mutations in the tissue of interest Epigenetic status in tissue of interest	qPCR NGS	Not limited to a certain tissue Minimal invasive sampling	Only limited gene expression information No information about tissue of origin
ccfDNA [6,18,67,70]	Liquid biopsies (body fluids): blood, urine, saliva, i.a.	Mutations throughout the whole body	qPCR NGS	Minimal invasive sampling	No expression information available Fragmented
CTCs [6,7,18]	Liquid biopsies: blood, urine	Metastatic tumor cells	Flow Cytometry (RT-)qPCR NGS	Minimal invasive sampling Tumor DNA, RNA and proteins combined	Less useful in later tumor stages Limited to tumor diagnosis
